# DeepLoc 2.0: multi-label subcellular localization prediction using protein language models

**DOI:** 10.1093/nar/gkac278

**Published:** 2022-04-30

**Authors:** Vineet Thumuluri, José Juan Almagro Armenteros, Alexander Rosenberg Johansen, Henrik Nielsen, Ole Winther

**Affiliations:** Indian Institute of Technology Madras, Chennai 600036, India; Novo Nordisk Foundation Center for Protein Research, Faculty of Health and Medical Sciences, University of Copenhagen, Copenhagen 2200, Denmark; Department of Genetics, Stanford University School of Medicine, Stanford 94305, CA, USA; Department of Computer Science, Stanford University, Stanford 94305, CA, USA; Department of Genetics, Stanford University School of Medicine, Stanford 94305, CA, USA; Section for Bioinformatics, Department of Health Technology, Technical University of Denmark, Kongens Lyngby 2800, Denmark; Center for Genomic Medicine, Rigshospitalet (Copenhagen University Hospital), Copenhagen 2100, Denmark; Department of Biology, Bioinformatics Centre, University of Copenhagen, Copenhagen 2200, Denmark; Section for Cognitive Systems, Department of Applied Mathematics and Computer Science, Technical University of Denmark, Kongens Lyngby 2800, Denmark

## Abstract

The prediction of protein subcellular localization is of great relevance for proteomics research. Here, we propose an update to the popular tool DeepLoc with multi-localization prediction and improvements in both performance and interpretability. For training and validation, we curate eukaryotic and human multi-location protein datasets with stringent homology partitioning and enriched with sorting signal information compiled from the literature. We achieve state-of-the-art performance in DeepLoc 2.0 by using a pre-trained protein language model. It has the further advantage that it uses sequence input rather than relying on slower protein profiles. We provide two means of better interpretability: an attention output along the sequence and highly accurate prediction of nine different types of protein sorting signals. We find that the attention output correlates well with the position of sorting signals. The webserver is available at services.healthtech.dtu.dk/service.php?DeepLoc-2.0.

## Graphical Abstract

**Graphical Abstract:**
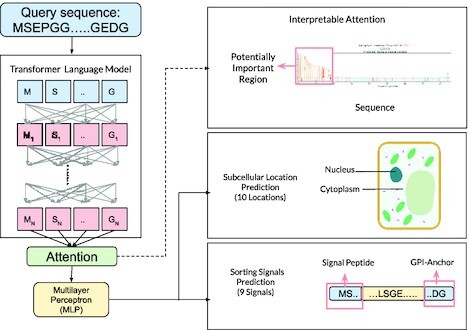
DeepLoc 2.0 uses a transformer-based protein language model to predict multi-label subcellular localization and provides interpretability via the attention and sorting signal prediction.

## INTRODUCTION

Identifying protein localization in different cellular compartments plays a key role in functional annotation. It can also aid in identifying drug targets (1), and understanding diseases linked to aberrant subcellular localization (2,3). Some proteins are known to localize in multiple cellular compartments (4–6). Several biological mechanisms have been identified to explain the localization process, which involves short sequences known as sorting signals (7–10).

Several machine learning-based methods exist for predicting subcellular localization. They can vary in the output prediction, i.e. single versus multi-location, or in the input features. YLoc+ (11) predicts multiple locations using biological features such as sorting signals, PROSITE (http://prosite.expasy.org/) patterns and optionally Gene Ontology (GO) terms from a database. Fuel-mLoc (12) on the other hand uses only GO terms from a custom database called ProSeq-GO to predict multiple locations for a variety of organisms. DeepLoc 1.0 (13) and LAProtT5 (14) predict a single location based on features extracted from only the sequence (sequence profiles in the case of DeepLoc) using deep learning models.

DeepLoc 1.0 uses a three stage deep learning approach for sequence classification. First, a feature representation for each amino acid in the sequence is generated. Then an attention-based pooling stage produces a single representation for the whole sequence. Finally, the prediction stage uses a classifier to output the subcellular labels.

DeepLoc 2.0 uses the same template while updating important aspects:

- Dataset: We curate large strict homology partitioned datasets of eukaryotic (15) and human proteins (16) for training and independent testing. We also compiled a dataset with experimentally verified annotation of nine types of sorting signals.
- Feature representation: We use a pre-trained protein transformer language model.
- An attention plot visualizes what part of the input the model uses for its predictions. Thus pointing to regions responsible for localization and potentially containing sorting signals. We use supervised learning with regularization to improve the interpretability of the attention plot.
- Prediction stage: We predict multiple labels for both the ten class subcellular localization and nine class sorting signals tasks.

## WEBSERVER

**Figure 1.**
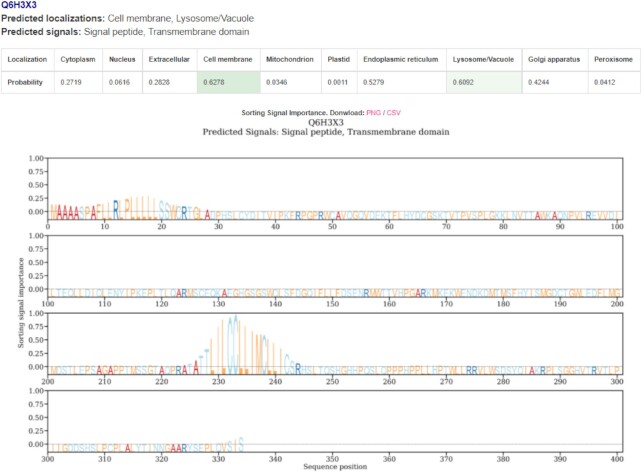
An example snippet from the results page on the webserver. The prediction summary is available for download as a comma-separated file (CSV) at the top which consists of the predicted subcellular localization and sorting signals. The image or attention values of each plot can be separately downloaded. All the predicted subcellular localization and sorting signal labels are listed, along with the prediction score table. The predicted localizations in the table are highlighted in green. If no score crosses the threshold, the label closest to the threshold is chosen. High values in the logo-like plot signify important regions in the sequence for localization prediction that may correspond to sorting signals. This is meant to serve as a guideline and specialized tools such as SignalP or TargetP can be used for a more detailed and accurate analysis of these signals.

The webserver is free and open to all and there is no login requirement. It takes in a maximum of 500 input sequences in the FASTA format. The model’s attention is shown in a figure when the long result format is toggled. Regions with high attention values are used by the model for its prediction and they are indicative of the presence of sorting signals. Once the job is submitted, it enters a queue and a waiting page is shown. The users can provide an email address to be notified of the results or the page automatically redirects when the results become available. An example prediction page is shown in Figure 1. Note that our model provides an output regardless of the input sequence. However, it is very difficult for us to judge whether a prediction is sensible if it is not a eukaryotic protein. Detailed estimate of prediction times is provided in Supplementary Table S1.

## DATA

We curate three datasets: two datasets with subcellular localization labels for cross-validation and independent validation, respectively, and a third dataset consisting of sorting signal labels, both the presence and location within the sequence, which is a part of the cross-validation dataset. Detailed statistics regarding the distribution of subcellular localization labels in the datasets are provided in Supplementary Figure S1 (Figure inspired by (17)).

### SwissProt localization dataset

The protein data were extracted from the UniProt database release 2021_03 (15). The protein sequences and localization annotations were then filtered using the following criteria: eukaryotic, not fragments (these could have N-terminal or C-terminal sorting signals missing), encoded in the nucleus, >40 amino acids and experimentally annotated (ECO:0000269) subcellular localizations. These proteins can be categorized into one or multiple of these ten locations: Cytoplasm, Nucleus, Extracellular, Cell membrane, Mitochondrion, Plastid, Endoplasmic reticulum, Lysosome/Vacuole, Golgi apparatus, Peroxisome. The details of the sublocation mapping and the number of proteins in each category are provided in Supplementary Table S2. This dataset is used for 5-fold cross-validation after homology-based partitioning (Supplementary Section S1).

### Human protein atlas

The Human Protein Atlas (HPA) project provides subcellular localization of human proteins using confocal microscopy (16). The annotations are provided with four reliability labels: Enhanced, Supported, Approved, and Uncertain, based on various criteria such as antibody validation and experimental evidence in the literature. We consider only Enhanced and Supported annotations for our independent test set since these are the most reliable labels. This dataset is ensured to not have any sequences with a >30% global sequence identity with the Swissprot Localization dataset described above and is used for independent validation.

### Sorting signals

Annotated sorting signals that are experimentally verified were mainly compiled from the literature. Supplementary Table S3 is a list of signals and their sources. We excluded proteins that were not present in our constructed SwissProt Localization Dataset. This dataset is used in the cross-validation procedure.

## DEEPLOC 2.0 OVERVIEW

**Figure 2.**
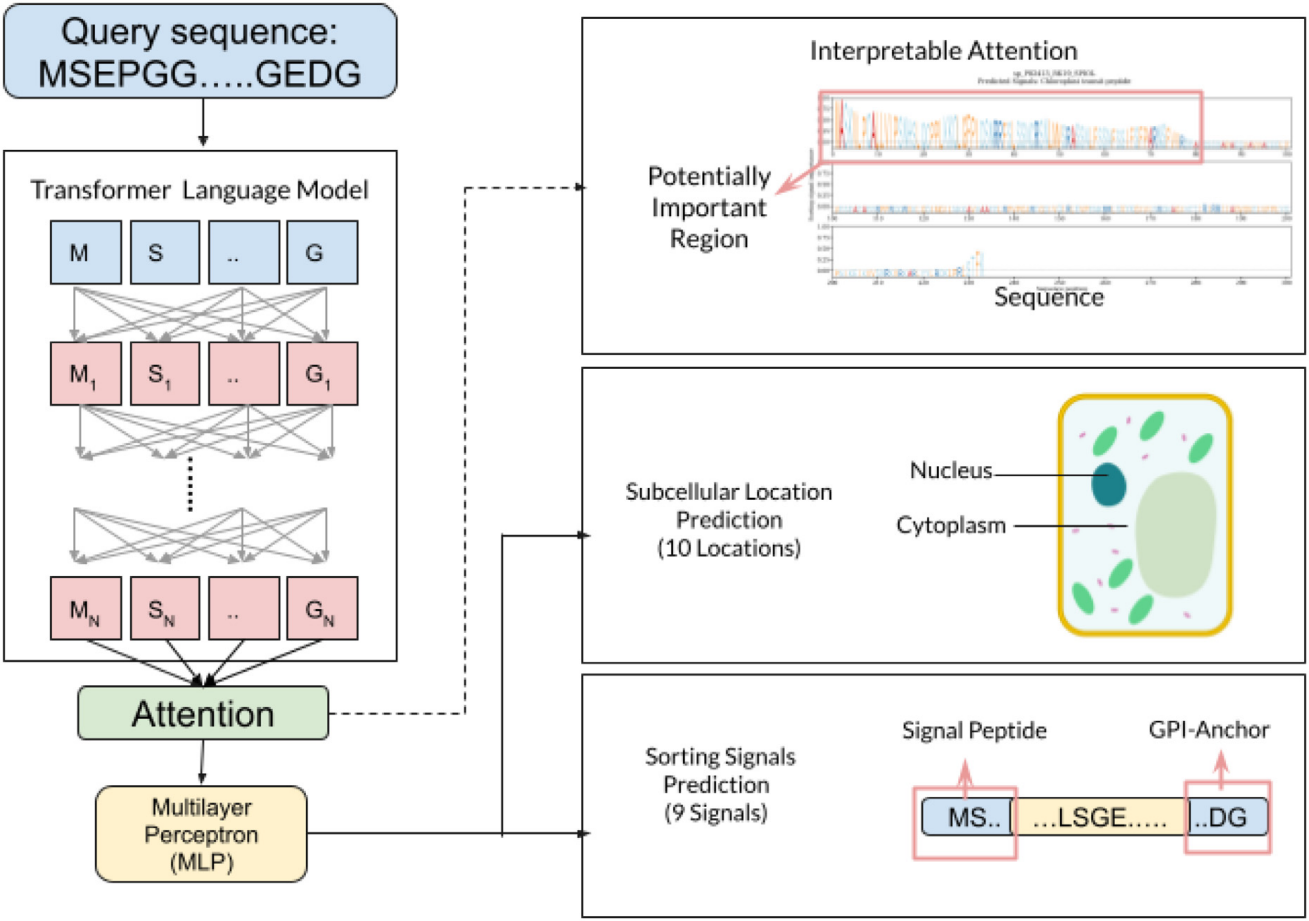
DeepLoc 2.0 uses a transformer-based protein language model to encode the input amino acid sequence. Then using an interpretable attention pooling mechanism a sequence representation is produced. The two prediction heads then utilize this representation to predict multiple labels for both the 10-type subcellular localization and 9-type sorting signal prediction tasks. Source of cell diagram: https://commons.wikimedia.org/wiki/File:Simple_diagram_of_plant_cell_(blank).svg, attribution: domdomegg, CC BY 4.0 <https://creativecommons.org/licenses/by/4.0>, via Wikimedia Commons.

As shown in Figure 2, the method can be broadly divided into three stages, each of which is briefly described below. More detailed information can be found in the Supplementary Section S2.

### Per-token representation using a transformer model

We utilize transformer-based language models (18) that have been successfully applied to the protein domain due to the abundance of unlabelled raw sequence data. They are trained in a self-supervised fashion on a large corpus, such as the UniRef50 database (19), using the masked language-modelling objective (20). The transformer is a deep learning method that uses multiple layers of the self-attention mechanism to produce representations that have been found to encode contact maps, taxonomy, and biophysical characteristics in their distributed representations (21–27). We evaluated three publicly available transformer models, the 12-layer ESM (Evolutionary Scale Modelling, (21)) model with 84M parameters, the 33-layer ESM model with 650M parameters (23) and the 3B parameter ProtT5-XL-UniRef50 model (24), referred to as ESM12, ESM1b and ProtT5, respectively, throughout the rest of the manuscript. The output of the language model is a vector representation for each residue (token) in the input sequence.

### Sequence representation using attention pooling

The per-token representations are combined using attention (28): First, a scalar score is computed for each token by taking the dot-product of the representation with a learnable vector. The learnable vector is tuned using supervised learning by using both the subcellular localization labels as well as the sequence annotation of the sorting signals. We smooth the scalar scores along the sequence by applying a 1d Gaussian filter of width 5, clipped at one standard deviation, to account for signals being present in a contiguous set of residues. The attention weights over the sequence are then computed using the softmax function on the smoothed scores so that they sum to 1. The output representation is the attention weighted sum of the token representations. This attention pooled representation vector is used as input to the prediction stage. The attention weights, visualized in the webserver, and the prediction of the sorting signals provide a better understanding of the predictions of the model.

### Multi-label localization and signal type prediction

The prediction stage consists of two multi-layer perceptron (MLP) classifier heads. The first head is trained along with the learnable vector from the attention step for the ten-class multi-label subcellular localization task. A second head is trained after freezing the rest of the parameters for the nine-class sorting signal prediction task. We found that optimizing for both tasks simultaneously proved to be difficult, hence we trained them one after another. These classifiers output a probability for each label. A weighted focal loss (29) is used for each label independently and then the losses for all labels are averaged so that they are jointly optimized. A threshold for each output label is computed by maximizing Matthew’s Correlation Coefficient (MCC) (30) on the training data. Accuracy-based metrics are susceptible to imbalance (31) that the MCC metric can handle better. Both these predictions are provided as outputs.

## RESULTS AND DISCUSSION

We chose YLoc+, DeepLoc 1.0, Fuel-mLoc, and LAProtT5 tools for comparison. These tools have public webservers or easily available local implementations. Since the outputs are different for each of the methods, we map the locations to the ten classes used in this work. We also reduce the Fuel-mLoc database by about 2% to remove close homologs to the test set for a fair comparison. The details of the mappings are provided in Supplementary Tables S4 and S5, modifications to the methods are described in the Supplementary Section S3. Additionally, in Supplementary Section S4, we provide insights from our experiments that the reader might find useful.

### Multi-label classification results

On the cross-validation dataset (Table 1), DeepLoc 2.0 has the highest scores in all metrics. Details of the model performance based on the kingdom of the protein are provided in Supplementary Table S10.

**Table 1.** Results on the SwissProt CV dataset

	Counts	DeepLoc 1.0 ^β^	YLoc+ ^α^	DeepLoc 2.0	
				ESM1b	ProtT5
Type		Single	Multi	Multi	Multi
Pred. Num. Labels (Actual: 1.27)		1.00 ± 0.00	1.57 ± 0.02	1.27 ± 0.02	1.26 ± 0.02
Accuracy	28303	0.48 ± 0.01	0.32 ± 0.02	0.53 ± 0.02	**0.55 ± 0.02**
Jaccard	28303	0.56 ± 0.01	0.50 ± 0.01	0.68 ± 0.01	**0.69 ± 0.01**
MicroF1	28303	0.58 ± 0.02	0.56 ± 0.01	0.72 ± 0.01	**0.73 ± 0.01**
MacroF1	28303	0.47 ± 0.01	0.42 ± 0.01	0.64 ± 0.01	**0.66 ± 0.01**
MCC per location (↑ is better)					
Cytoplasm	9870	0.45 ± 0.02	0.38 ± 0.02	0.61 ± 0.01	**0.62 ± 0.01**
Nucleus	9720	0.46 ± 0.02	0.42 ± 0.02	0.66 ± 0.02	**0.69 ± 0.01**
Extracellular	3301	0.78 ± 0.05	0.61 ± 0.05	**0.85 ± 0.03**	**0.85 ± 0.04**
Cell membrane	4187	0.53 ± 0.02	0.44 ± 0.02	0.64 ± 0.01	**0.66 ± 0.01**
Mitochondrion	2590	0.58 ± 0.04	0.47 ± 0.02	0.73 ± 0.03	**0.76 ± 0.02**
Plastid	1047	0.69 ± 0.04	0.72 ± 0.02	0.88 ± 0.01	**0.90 ± 0.01**
Endoplasmic reticulum	2180	0.32 ± 0.04	0.17 ± 0.04	0.52 ± 0.01	**0.56 ± 0.03**
Lysosome/Vacuole	1496	0.06 ± 0.05	0.07 ± 0.03	0.24 ± 0.03	**0.28 ± 0.04**
Golgi apparatus	1279	0.20 ± 0.04	0.11 ± 0.04	**0.36 ± 0.06**	0.34 ± 0.05
Peroxisome	304	0.15 ± 0.04	0.05 ± 0.02	0.48 ± 0.05	**0.56 ± 0.08**

**Bold** values indicate the best score

^α^ = GO-terms were not used

^β^ = Retrained on this dataset

From Table 2, on the independent HPA benchmark, we find that DeepLoc 2.0 outperforms other tools on several metrics except for the accuracy and MCC for nucleus which are highest for the LAProtT5 method. The MCC for endoplasmic reticulum is highest for the DeepLoc 1.0 method. DeepLoc 2.0 predicts a realistic average number of labels per protein compared to other methods. Supplementary Table S6 contains the results for all the methods and variants we benchmarked on this dataset. Supplementary Table S7 contains a threshold-independent comparison using the Area under the ROC (AUC) metric for methods which also output a prediction score.

**Table 2.** Results on the HPA independent test set

	Count	YLoc+	DeepLoc 1.0 ^β^	Fuel-mLoc	LAProtT5	DeepLoc 2.0	
		Animal^α^		Euk ^γ, θ^		ESM1b	ProtT5
Type		Multi	Single	Multi	Single	Multi	Multi
Pred. Num. Labels (Actual: 1.22)		1.44	0.89	1.00	0.94	1.15	1.21
Accuracy	1717	0.23	0.37	0.38	**0.45**	0.34	0.39
Jaccard	1717	0.41	0.42	0.46	0.52	0.48	**0.53**
MicroF1	1717	0.51	0.46	0.52	0.56	0.57	**0.60**
MacroF1	1717	0.34	0.35	0.39	0.43	0.44	**0.46**
MCC per location (↑ is better)							
Cytoplasm	562	0.14	0.23	0.23	0.33	0.29	**0.36**
Nucleus	893	0.20	0.28	0.41	**0.45**	0.41	0.44
Cell membrane	287	0.20	0.23	0.32	0.30	0.34	**0.36**
Mitochondrion	196	0.37	0.39	0.33	0.59	**0.60**	0.56
Endoplasmic reticulum	77	0.12	**0.23**	0.14	0.22	0.20	0.17
Golgi apparatus	86	0.08	0.10	0.24	0.26	0.17	**0.31**

**Bold** values indicate the best score

^α^ = GO-terms were not used

^β^ = Retrained on the new CV dataset

^γ^ = using local implementation

^θ^ = using reduced ProSeq database

### Sorting signal prediction results

#### Signal type prediction

Table 3 shows that DeepLoc 2.0 is able to distinguish between the nine signal types in most of the cases with high accuracy (79%). The worst performance is obtained for nuclear export signals. Additionally, the table shows the performances we measured on the sorting signals dataset by three specialized predictors: SignalP 6.0 (32) for signal peptides, TargetP 2.0 (33) for mitochondrial and plastid transit peptides, and NetGPI 1.1 (34) for GPI anchors. Note that some of the sequences in the sorting signals dataset may have been included in the training sets of the specialized predictors, while the DeepLoc 2.0 values are cross-validated. DeepLoc 2.0 shows state-of-the-art performance in recognizing signal peptides and mitochondrial transit peptides. However, the specialized tools must be consulted in order to obtain the exact lengths of the sorting signals.

**Table 3.** Results of signal type prediction; cross-validation

	DeepLoc 2.0		Specialized
	ESM1b	ProtT5	Predictor
MicroF1	0.87 ± 0.01	0.87 ± 0.02	
MacroF1	0.80 ± 0.02	0.80 ± 0.03	
Accuracy	0.78 ± 0.02	0.79 ± 0.03	
MCC per signal (↑ is better)			
SP	0.89 ± 0.03	0.90 ± 0.03	0.87 ± 0.02 (32)
TM	0.71 ± 0.07	0.66 ± 0.05	-
MT	0.93 ± 0.02	0.93 ± 0.03	0.94 ± 0.04 (33)
CH	0.85 ± 0.07	0.86 ± 0.09	0.96 ± 0.03 (33)
TH	0.86 ± 0.08	0.80 ± 0.08	0.98 ± 0.04 (33)
NLS	0.65 ± 0.06	0.66 ± 0.01	-
NES	0.49 ± 0.20	0.46 ± 0.17	-
PTS	0.85 ± 0.06	0.90 ± 0.05	-
GPI	0.85 ± 0.06	0.86 ± 0.06	0.91 ± 0.01 (34)

SP = Signal Peoptide, TM = First transmembrane domain, MT = Mitochondrial transit peptide , CH = Chloroplast transit peptide, TH = Thylakoidal transit peptide, NLS = Nuclear localization signal, NES = Nuclear export signal, PTS = Peroxisomal targeting signal, GPI = GPI-anchor

#### Attention-signal correlation

Table 4 demonstrates that DeepLoc 2.0’s attention is far better than that of DeepLoc 1.0 at providing insights into the sorting signals. The Kullback–Leibler (KL) divergence, a direct measure of dissimilarity between attention and signal, is lower for DeepLoc 2.0. More detailed metrics and comparisons are available for each sorting signal in the Supplementary Tables S8 and S9.

**Table 4.** Quantitative comparison of interpretable attention; cross-validation

	DeepLoc 1.0 ^β^	DeepLoc 2.0	
		ESM1b	ProtT5
KL Div (↓ is better)			
SP	1.31 ± 0.57	1.04 ± 0.91	0.99 ± 0.86
TM	1.99 ± 0.81	1.13 ± 1.14	1.12 ± 1.03
MT	0.92 ± 0.38	0.51 ± 0.54	0.50 ± 0.48
CH	0.74 ± 0.33	0.32 ± 0.52	0.31 ± 0.31
TH	0.90 ± 0.31	0.19 ± 0.29	0.24 ± 0.16
NLS	3.11 ± 1.02	2.63 ± 1.52	2.60 ± 1.32
NES	3.97 ± 1.22	4.04 ± 1.51	3.88 ± 1.44
PTS	4.90 ± 0.93	0.85 ± 1.29	0.72 ± 1.05
GPI	2.30 ± 0.79	1.59 ± 0.73	1.85 ± 0.47

^β^ = Retrained on the new CV dataset

Abbreviations same as in Table 3

## CONCLUSION

We provide a multi-label subcellular localization prediction tool, based on protein language models, that uses only the sequence information and outperforms existing methods. This is made possible by the use of a large curated dataset with annotations of multi-location proteins. Additionally, using a small dataset of sorting signals, we were able to improve the interpretability of the attention layer in our model. Thus, we can also provide the predicted signal type and important regions, which can give insights into relevant sections of the protein sequence that are responsible for particular localization. The webserver is available at https://services.healthtech.dtu.dk/service.php?DeepLoc-2.0.

## DATA AVAILABILITY

The data used for training and testing are available at https://services.healthtech.dtu.dk/service.php?DeepLoc-2.0.

## Supplementary Material

gkac278_Supplemental_File
