# Supplementary material for: DeepLoc 2.0: multi-label subcellular localization prediction using protein language models
Source: Nucleic Acids Res. 2022 Apr 30;50(W1):W228–34. doi: 10.1093/nar/gkac278 (PMC9252801; doi:10.1093/nar/gkac278)
Supplement: gkac278_Supplemental_File [file gkac278_supplemental_file.pdf]

SUPPLEMENTARY MATERIAL FOR DEEPLOC 2.0

Supplementary Table 1. Webserver estimated time per sequence. Note that the model load time is constant for any number of input sequences, whereas the prediction and plot time scale linearly with the number of sequences.

	ESM1b	ProtT5
Short sequences (Average length: 104)		
Model load time (s)	11.07	26.80
Prediction time (s / seq)	0.83	3.93
Plot time (s / seq)	2.38	2.57
Long sequences (Average length: 400)		
Model load time (s)	11.09	26.09
Prediction time (s / seq)	3.29	7.33
Plot time (s / seq)	7.94	7.97

1 DATA PARTITIONING

To generate high-quality data partitions for the SwissProt dataset, we adopted the procedure described by Gíslason et al. (7) to generate label-balanced splits for 5-fold cross-validation. This procedure ensures that each pair of train and test fold does not share sequences that have global sequence identity greater than 30% as determined using ggsearch36, which is a part of the FASTA package (8).

The Human Protein Atlas (9) project provides annotation for 78,136 proteins. The HPA independent dataset was constructed using the following steps:

1. Homology reduction to ensure a maximum of 30% sequence identity to the whole SwissProt dataset using USEARCH v11.0.667, 32-bit (10). This leaves 23,422 proteins.
2. Selection of only ”Enhanced” or ”Supported” annotations to improve the reliability of labels. This leaves 5,523 proteins,
3. Clustering of sequences with 90% identity threshold and selecting the centroids using USEARCH v11.0.667, 32-bit, to reduce measuring correlated errors. Also Removing Peroxisome and Lysosome/Vacuole localizations since they are few in number. This leaves 2445 proteins
4. Additionally, we remove sequences that have a greater than 30% sequence identity to any sequence from the multi-label eukaryote dataset (11), which was used to train Fuel-mLoc. This leaves 1,717 proteins.

2 DEEPLOC 2.0: IMPLEMENTATION DETAILS

2.1 Transformer models:

We use three publicly available transformer models, the 12-layer ESM (Evolutionary Scale Modelling, 12) model with 84M parameters, the 33-layer ESM model with 650M parameters (13), and the 3B parameter ProtT5-XL-UniRef50 model (14), referred to as ESM12, ESM1b, and

Supplementary Figure 1. The subcellular localization statistics of the cross-validation and independent test datasets.

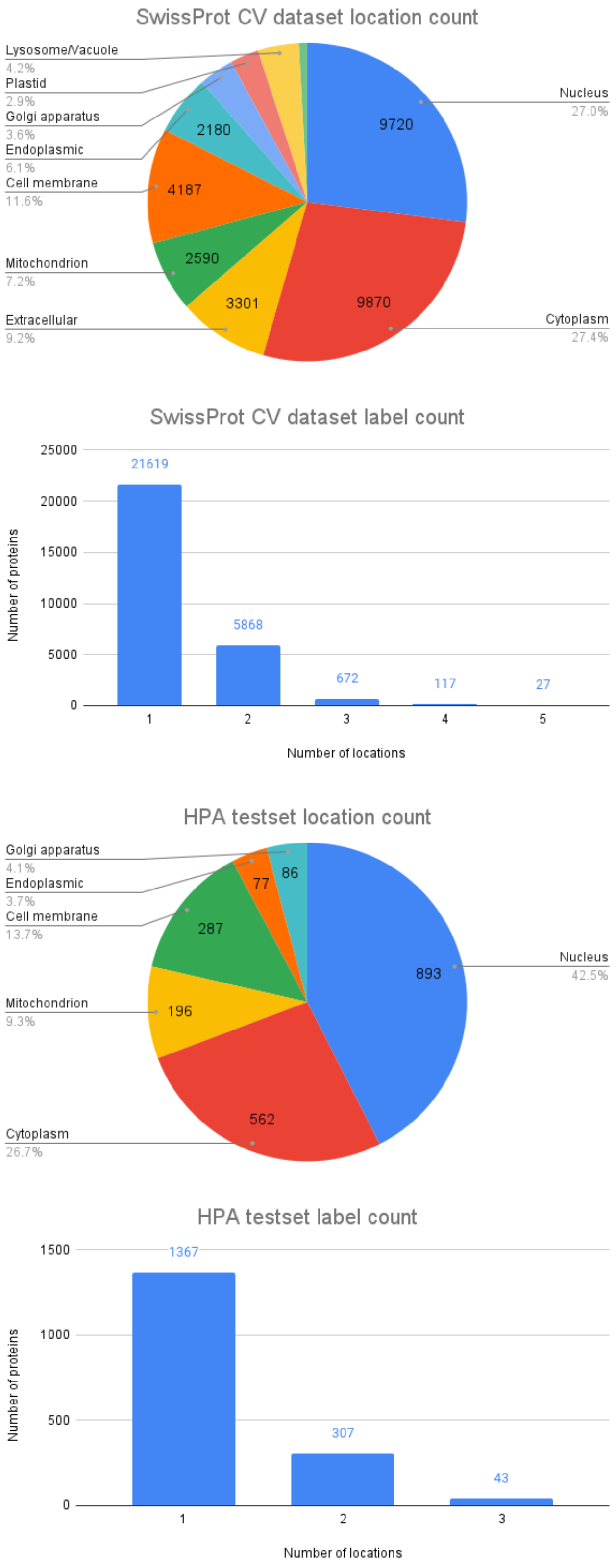

Supplementary Table 2. SwissProt cross-validation dataset: Number of proteins in each location and sublocations that were grouped together under the same main location

Location	No. of proteins	Sublocations
Nucleus	9720	Envelope, inner and outer membrane, matrix, lamina, chromosome, nucleus speckle
Cytoplasm	9870	Cytoplasm (cytosol and cytoskeleton)
Extracellular	3301	Extracellular
Mitochondrion	2590	Envelope, inner and outer membrane, matrix, intermembrane space
Cell membrane	4187	Apical, apicolateral, basal, basolateral, lateral, cell membrane, cell projection
Endoplasmic reticulum (ER)	2180	ER membrane and lumen, microsome, rough ER, smooth ER, Sarcoplasmic reticulum
Plastid	1047	Plastid membrane, stroma and thylakoid
Golgi apparatus	1279	Golgi apparatus membrane and lumen
Lysosome/Vacuole	1496	Contractile, lytic and protein storage vacuole, vacuole lumen and membrane, lysosome lumen and membrane
Peroxisome	304	Peroxisome matrix and membrane

Supplementary Table 3. Sorting signals dataset: Signal annotations and their sources from the literature

Signal	Count	Source
Signal Peptides (SP)	1011	(1), (2) and (3)
Transmembrane domains (TM)	260	(2) (Note that we only use the first domain which has been shown to direct localization (4, 5))
Mitochondrial transit peptide (MT)	242	(3)
Chloroplast transit peptide (CH)	90	(3)
Thylakoidal lumen composite transit peptide (TH)	42	(3)
Nuclear localization signal (NLS)	148	(6)
Nuclear export signal (NES)	100	(6)
Peroxisome targeting signal (PTS)	127	We filtered the SwissProt motif annotations to contain either ”peroxisome”, ”peroxisomal” or ”microbody” in their description.

ProtT5 respectively throughout the rest of the manuscript. Additionally a suffix ”(S)” or ”(M)” is added to indicate whether the model was trained with single or multi-location labels.

The maximum sequence length used for training was 1022 for the ESM models and 4000 for ProtT5. Proteins that exceeded this length had the middle portion of their sequence removed so that the ends are retained. This value is chosen to be lower for ESM models because of the limitations of the ESM1b model architecture.

2.2 Multi-label prediction loss

We used weighted focal loss (15) with the binary cross-entropy objective to train the location predictions. The weight of each of the ten localization labels was set to be inversely proportional to the label frequency in the training dataset. This is done so that all labels are represented equally in the loss. The γ parameter of the focal loss is set to 1 following previous works that use similar losses for multi-label classification (16, 17).

$$p_l = yp + (1-y)(1-p) \quad (1)$$

$$L_{ML} = \sum_{l \epsilon L} -w_l(1-p_l)^{\gamma} log(p_l) \quad (2)$$

where L is the set of all labels, y is the target label, w_l is the weight for the label, p is the output probability for the label, and γ is focal loss parameter.

2.3 Supervision using sorting signals

Normalized KL-divergence loss between the attention and the annotated signal is used whenever available. Additionally, we weight the loss to make the effective number of samples of each signal type the same.

$$L_{KL} = \sum_{i}^{L} p_i log(\frac{p_i}{q_i}) \quad (3)$$

where L is the length of the full sequence, q is the attention. p is the target probability distribution which is defined as follows, it is 0 in positions where the sorting signal is not present and $\frac{1}{L_p}$ otherwise, where L_p is the length of the sorting signal.

2.4 DCT-prior-based regularization

In previous work, Tseng et al. (18) regularized the saliency (input times gradient) based on the Fourier transform and found that this improves the interpretability and stability of training by increasing the signal-to-noise ratio. We use this

idea for our attention-pooling layer instead. It is known that some sorting signals are present at the N- and C- termini and thus we expect the learned attention to mimic this. Since the Fourier transform is computed assuming the periodicity of the signals, discontinuities at the ends of the sequence lead to artifacts. We avoid this issue by replacing the Fourier transform with the Discrete-Cosine Transform (DCT).

We first smooth the raw attentions scores (before softmax) using a gaussian 1-d convolutional filter of size 5 clipped to one standard deviation (K). The attention is then padded on both sides by repeating the value at the ends. The regularization loss is then computed by adding up the coefficients using the following weighting scheme. The first $\frac{L}{6}$ normalized DCT coefficients have the weight 1, and the rest are weighted using an exponentially decaying function $\frac{1}{1+x^{0.2}}$. Here, L is the length of the sequence and x is the index of the coefficient subtracted by $\frac{L}{6}$.

$$A = Softmax(K*S) \tag{4}$$

$$D = \frac{|DCT(A)|}{\sum |DCT(A)|} \tag{5}$$

$$L_{DCT} = \sum_{i}^{L} w_i D_i \tag{6}$$

where L is the length of the full sequence, S is the raw attention scores. K is the gaussian kernel described above, $*$ operation represents convolution. w_i's are the weights according to the scheme described above.

$$L = L_{ML} + 0.1 L_{KL} + 0.1 L_{DCT} \tag{7}$$

The final loss is a weighted combination of the three losses. The attention supervision loss and the regularization loss are scaled by 0.1 to ensure that the secondary losses do not dominate the multi-label loss.

2.5 Signal type prediction

Since optimizing over multiple tasks is quite challenging, we freeze the model parameters after training with the multi-label localization and sorting signals prediction objectives. The pooled embedding vector after the attention and the final prediction probabilities are used as the input to a Multi-layer perceptron (MLP) to predict signal types in a multi-label fashion.

2.6 Training details

Different learning rates for the transformer encoder (5×10^{-6} if finetuning, 0 otherwise) and the attention-pooling, classification layer (5×10^{-5}) were used. The training was terminated after a fixed number of epochs to ensure that the models always overfit based on the randomly sampled validation set. The max number of epochs was 5 in the case of finetuning and 15 for the frozen models. The final model was picked by taking the best validation loss over all epochs. Mixed-precision and model sharding techniques were utilized to efficiently fine-tune the models. The PyTorch-lightning (19) library and hardware provided by Google Colaboratory GPUs[1], and 2 Tesla V100s were used for training and testing.

3 EXPERIMENTS

3.1 Benchmarking existing methods

For our experiments, DeepLoc 1.0, a retrained version of DeepLoc 1.0, and ESM12 (S) are the single-location predictors used as a baseline. The rest predict multiple locations. Each method considers a different set of possible locations, therefore we map each of these to the locations defined in Section 2.1. After reduction, the HPA dataset either does not contain or has very few proteins in Extracellular, Plastid, Lysosome/Vacuole, and Peroxisome locations and so we exclude these locations from the true labels. A consequence of this is that single label predictors can have an average number of predicted labels to be less than one since the predictions can fall outside these six true labels on the HPA independent test set.

Sequence-based methods: For YLoc+, we downloaded the standalone version of the predictor and used the animal, plant or fungi predictor whichever was appropriate, without the GO-terms option. DeepLoc-1.0 was retrained using the same procedure as originally described in (20).

GO-based methods: Sequence-based methods can be easier to benchmark against since proper homology-partitioning between the training and test sets ensures that the performance evaluation is a good estimate for unseen proteins. On the other hand, GO-based methods rely on local-alignment scores using a large database of indirect localization labels i.e. GO terms. Since the partitioning is only done considering the training and test sets and not the database, there is potential homology ”leakage”. This can lead to performance overestimation of these methods. We found that about $\sim 93\%$ of sequences after Step 4 in Section 3.1 have a sequence-identity match of greater than 30% with the ProSeq-GO database. Therefore, we reimplement Fuel-mLoc, changing only the database used. Based on the global sequence identity measured using USEARCH, we exclude 10,550 sequences (about 2% of the database), from the BLAST search. To ensure a fair comparison, first we consider Fuel-mLoc, for which the webserver was used to obtain the predictions, by selecting ”Eukaryotes” and ”Local database” in the options. Then, we reimplement it without any changes to the database to confirm that our version is as close to the original as possible. Finally, we use the reduced database to produce the results for comparison. Detailed results are provided in Supplementary Table 5.

The mapping of locations for the methods used in the comparison is provided in Supplementary Table 2 and 3.

3.2 Metrics for subcellular localization

We use the following metrics to comprehensively quantify the classification performance on the datasets:

[1]https://colab.research.google.com/

- Number of predicted labels: Averaged over all predictions, this demonstrates the bias of the predictor.
- Accuracy: Requires the exact location(s) to be predicted. Since the dataset is skewed towards proteins with single localization, this metric provides an advantage to single-label predictors.
- Jaccard: Overlap between the actual and predicted labels over their union.
- MicroF1: F1 score considering the total number of true positives, false negatives, and false positives.
- MacroF1: F1 score computed for each class and then averaged, providing equal emphasis on rare and frequent classes.
- Matthews Correlation Coefficient: Measured for each class, it requires the model to perform well on all four confusion matrix entries.

For our multi-label prediction models, we computed the thresholds for each class by maximizing the MCC on the training set. The prediction thresholds can be found on the output page after submitting proteins to the webserver.

3.3 Measuring the interpretability of attention

For quantifying the relevance of attention to the sorting signals, we use the following metrics:

- Importance in signal: Total attention mass present within the signal.
- Signal over background: The average attention value within the signal over the average value outside the signal.
- Metric Entropy: The entropy of the attention normalized by the information length of the protein. It ranges from 0 to 1, with lower values indicating that larger attention mass is placed on fewer residues.
- KL-Divergence: Distributional dissimilarity between the signal and attention.

4 RESULTS

4.1 Preliminary questions

Improvements due to transformer models: We trained ESM12 (Single) and retrained DeepLoc-1.0 on our cross-validation dataset, but using only proteins with a single location. From Table 6, we find that ESM12 (Single) outperforms both DeepLoc-1.0 and its retrained version significantly. This is to be expected since ESM12 (Single) is a much larger model with unsupervised pretraining on a large dataset.

Improvements due to multi-label annotations: Comparing ESM12 (Single) and ESM12 (Multi) we see that while the absolute accuracy has dropped, the rest of the metrics show that the predictions are indeed better overall, as well as for each location. Thus we conclude that the multi-label annotations provide additional useful information to the models.

Supplementary Table 4. YLoc to DeepLoc mapping

YLoc Location	DeepLoc location
nucleus	Nucleus
mitochondrion	Mitochondrion
plasma membrane	Cell membrane
extracellular space	Extracellular
Golgi apparatus	Golgi apparatus
lysosome	Lysosome/Vacuole
vacuole	Lysosome/Vacuole
chloroplast	Plastid
cytoplasm	Cytoplasm
peroxisome	Peroxisome
ER	Endoplasmic reticulum

Supplementary Table 5. Fuel-mLoc to DeepLoc mapping

Fuel-mLoc Location	DeepLoc location
Acrosome	Cytoplasm
Cell-Membrane	Cell membrane
Cell-Wall	Cell membrane
Chloroplast	Plastid
Cyanelle	Cytoplasm
Cytoplasm	Cytoplasm
Cytoskeleton	Cytoplasm
Endosome	Cytoplasm
Extracellular	Extracellular
Golgi-Apparatus	Golgi apparatus
Hydrogenosome	Cytoplasm
Lysosome	Lysosome/Vacuole
Centrosome	Cytoplasm
Endoplasmic-Reticulum	Endoplasmic reticulum
Melanosome	Cytoplasm
Microsome	None
Mitochondrion	Mitochondrion
Nucleus	Nucleus
Peroxisome	Peroxisome
Spindle-Pole-Body	Cytoplasm
Synapse	Extracellular
Vacuole	Lysosome/Vacuole

4.2 Loss term ablation

We trained the largest and smallest models with and without the two additional losses for the attention layer i.e. the supervision and regularization losses. We observed that the multi-label localization performance was mostly unaffected. However, the interpretability increases dramatically by including these loss terms. Supplementary tables 8, 9 show that when trained with these terms included, the signals are more prominent compared to the background, more of the attention is placed within the signal, the signal is sparser, and the KL-Divergence is lower implying that the attention correlates better with the signal.

Supplementary Table 6. HPA Dataset. Independent test set. Performance of all the methods

	YLoc+ -Animal$^{\alpha}$	DeepLoc-1.0 $^{\beta}$	DeepLoc-1.0	Fuel-mLoc Euk	Fuel-mLoc Euk $^{\gamma}$	Fuel-mLoc Euk $^{\gamma,\theta}$
Type	Multi	Single	Single	Multi	Multi	Multi
Pred. Num. Labels (Actual: 1.22)	1.44	0.89	0.90	1.03	1.06	1.00
ACC	0.23	0.37	0.38	0.48	0.48	0.38
Jaccard	0.41	0.42	0.44	0.56	0.57	0.46
MicroF1	0.51	0.46	0.47	0.61	0.62	0.52
MacroF1	0.34	0.35	0.35	0.55	0.55	0.39
Cytoplasm	0.14	0.23	0.22	0.33	0.35	0.23
Nucleus	0.20	0.28	0.33	0.53	0.53	0.41
Cell membrane	0.20	0.23	0.25	0.34	0.35	0.32
Mitochondrion	0.37	0.39	0.43	0.72	0.71	0.33
Endoplasmic reticulum	0.12	0.23	0.12	0.37	0.36	0.14
Golgi apparatus	0.08	0.10	0.17	0.44	0.45	0.24

	LAProtT5	ESM12	ESM1b $^{\delta}$	ESM1b	ProtT5 $^{\delta}$
Type	Single	Multi	Multi	Multi	Multi
Pred. Num. Labels (Actual: 1.22)	0.94	1.14	1.15	1.28	1.21
ACC	0.45	0.33	0.34	0.36	0.39
Jaccard	0.52	0.47	0.48	0.52	0.53
MicroF1	0.56	0.55	0.57	0.60	0.60
MacroF1	0.43	0.41	0.44	0.43	0.46
Cytoplasm	0.33	0.26	0.29	0.31	0.36
Nucleus	0.45	0.42	0.41	0.40	0.44
Cell membrane	0.30	0.32	0.34	0.31	0.36
Mitochondrion	0.59	0.57	0.60	0.67	0.56
Endoplasmic reticulum	0.22	0.17	0.20	0.09	0.17
Golgi apparatus	0.26	0.16	0.17	0.19	0.31

$^{\alpha}$ = GO-terms were not used
$^{\beta}$ = Retrained on the new CV dataset
$^{\gamma}$ = using local implementation
$^{\theta}$ = using reduced ProSeq database
$^{\delta}$ = Transformer parameters frozen

REFERENCES

1. Teufel, F., Almagro Armenteros, J. J., Johansen, A. R., Gíslason, M. H., Pihl, S. I., Tsirigos, K. D., Winther, O., Brunak, S., von Heijne, G., and Nielsen, H. (2022) SignalP 6.0 predicts all five types of signal peptides using protein language models. *Nature Biotechnology,*.
2. Tsirigos, K. D., Peters, C., Shu, N., Käll, L., and Elofsson, A. (2015) The TOPCONS web server for consensus prediction of membrane protein topology and signal peptides. *Nucleic Acids Research,* **43**(W1), W401–W407.
3. Almagro Armenteros, J. J., Salvatore, M., Emanuelsson, O., Winther, O., von Heijne, G., Elofsson, A., and Nielsen, H. (2019) Detecting sequence signals in targeting peptides using deep learning. *Life Science Alliance,* **2**(5), e201900429.
4. Kanner, E. M., Friedlander, M., and Simon, S. M. (2003) Co-translational Targeting and Translocation of the Amino Terminus of Opsin across the Endoplasmic Membrane Requires GTP but Not ATP. *Journal of Biological Chemistry,* **278**(10), 7920–7926.
5. Wang, J., Chen, J., Enns, C. A., and Mayinger, P. (2013) The First Transmembrane Domain of Lipid Phosphatase SAC1 Promotes Golgi Localization. *PLoS ONE,* **8**(8), e71112.
6. Bernhofer, M., Goldberg, T., Wolf, S., Ahmed, M., Zaugg, J., Boden, M., and Rost, B. (2018) NLSdb—major update for database of nuclear localization signals and nuclear export signals. *Nucleic Acids Research,* **46**(D1), D503–D508.
7. Gíslason, M. H., Nielsen, H., Almagro Armenteros, J. J., and Johansen, A. R. (2021) Prediction of GPI-anchored proteins with pointer neural networks. *Current Research in Biotechnology,* **3**, 6–13.
8. Pearson, W. R. and Lipman, D. J. (1988) Improved tools for biological sequence comparision. *Proc. Natl. Acad. Sci.,* **85**, 2444–2448.
9. Thul, P. J., Åkesson, L., Wiking, M., Mahdessian, D., Geladaki, A., Ait Blal, H., Alm, T., Asplund, A., Björk, L., Breckels, L. M., Bäckström, A., Danielsson, F., Fagerberg, L., Fall, J., Gatto, L., Gnann, C., Hober, S., Hjelmare, M., Johansson, F., Lee, S., Lindskog, C., Mulder, J., Mulvey, C. M., Nilsson, P., Oksvold, P., Rockberg, J., Schutten, R., Schwenk, J. M., Sivertsson, A., Sjöstedt, E., Skogs, M., Stadler, C., Sullivan, D. P., Tegel, H., Winsnes, C., Zhang, C., Zwahlen, M., Mardinoglu, A., Pontén, F., von Feilitzen, K., Lilley, K. S., Uhlén, M., and Lundberg, E. (2017) A subcellular map of the human proteome. *Science,* **356**(6340), eaal3321.
10. Edgar, R. C. (2010) Search and clustering orders of magnitude faster than BLAST. *Bioinformatics,* **26**(19), 2460–2461.
11. Chou, K.-C. and Shen, H.-B. (2010) A New Method for Predicting the Subcellular Localization of Eukaryotic Proteins with Both Single and Multiple Sites: Euk-mPLoc 2.0. *PLOS ONE,* **5**(4), 1–9.
12. Rives, A., Meier, J., Sercu, T., Goyal, S., Lin, Z., Liu, J., Guo, D., Ott, M., Zitnick, C. L., Ma, J., and Fergus, R. (2021) Biological Structure and Function Emerge from Scaling Unsupervised Learning to 250 Million Protein Sequences. *Proceedings of the National Academy of Sciences,* **118**(15), e2016239118.
13. Rao, R., Meier, J., Sercu, T., Ovchinnikov, S., and Rives, A. (2020)

Supplementary Table 7. AUC scores on the Cross-Validation and HPA independent test set for selected methods that output scores for predictions

	DeepLoc 1.0	DeepLoc 2.0 ESM1b	DeepLoc 2.0 ProtT5
AUC per location (↑ is better)			
Cross-validation dataset			
Cytoplasm	0.83 ± 0.01	0.88 ± 0.00	0.88 ± 0.01
Nucleus	0.86 ± 0.01	0.92 ± 0.01	0.93 ± 0.01
Extracellular	0.96 ± 0.01	0.98 ± 0.01	0.98 ± 0.01
Cell membrane	0.84 ± 0.02	0.92 ± 0.00	0.93 ± 0.01
Mitochondrion	0.90 ± 0.02	0.93 ± 0.01	0.93 ± 0.01
Plastid	0.97 ± 0.00	0.98 ± 0.00	0.99 ± 0.00
Endoplasmic reticulum	0.83 ± 0.03	0.89 ± 0.01	0.90 ± 0.02
Lysosome/Vacuole	0.72 ± 0.04	0.83 ± 0.03	0.85 ± 0.03
Golgi apparatus	0.76 ± 0.03	0.84 ± 0.03	0.85 ± 0.01
Peroxisome	0.83 ± 0.04	0.91 ± 0.03	0.91 ± 0.03

	Fuel-mLoc Euk	DeepLoc 1.0	DeepLoc 2.0 ESM1b	DeepLoc 2.0 ProtT5
HPA independent test set				
Cytoplasm	0.58	0.66	0.71	0.74
Nucleus	0.74	0.73	0.79	0.81
Cell membrane	0.64	0.67	0.76	0.78
Mitochondrion	0.71	0.82	0.87	0.88
Endoplasmic reticulum	0.54	0.64	0.70	0.75
Golgi apparatus	0.57	0.72	0.76	0.72

Transformer protein language models are unsupervised structure learners. *bioRxiv,* p. 2020.12.15.422761.

14. Elnaggar, A., Heinzinger, M., Dallago, C., Rihawi, G., Wang, Y., Jones, L., Gibbs, T., Feher, T., Angerer, C., Bhowmik, D., and Rost, B. (2021) ProtTrans: Towards Cracking the Language of Lifes Code Through Self-Supervised Deep Learning and High Performance Computing. *IEEE Transactions on Pattern Analysis and Machine Intelligence,* **14**(8).
15. Lin, T., Goyal, P., Girshick, R. B., He, K., and Dollár, P. (2017) Focal Loss for Dense Object Detection. *arXiv,* p. 1708.02002.
16. Liu, S., Zhang, L., Yang, X., Su, H., and Zhu, J. (2021) Query2Label: A Simple Transformer Way to Multi-Label Classification. *arXiv,* p. 2107.10834.
17. Ben-Baruch, E., Ridnik, T., Zamir, N., Noy, A., Friedman, I., Protter, M., and Zelnik-Manor, L. (2020) Asymmetric Loss For Multi-Label Classification. *arXiv,* p. 2009.14119.
18. Tseng, A., Shrikumar, A., and Kundaje, A. (2020) Fourier-transform-based attribution priors improve the interpretability and stability of deep learning models for genomics. In Larochelle, H., Ranzato, M., Hadsell, R., Balcan, M. F., and Lin, H., (eds.), *Advances in Neural Information Processing Systems*, Curran Associates, Inc. Vol. 33, pp. 1913–1923.
19. Falcon, W. et al. (2019) PyTorch Lightning. *GitHub. Note: https://github.com/PyTorchLightning/pytorch-lightning,* **3**.
20. Almagro Armenteros, J. J., Sønderby, C. K., Sønderby, S. K., Nielsen, H., and Winther, O. (2017) DeepLoc: prediction of protein subcellular localization using deep learning. *Bioinformatics,* **33**(21), 3387–3395.

Supplementary Table 8. Ablation of loss terms. DCT-based prior regularization and sorting signal supervision are indicated by Reg and Sup in short hand.

	ESM12		ProtT5	
	No Reg & Sup	With Reg & Sup	No Reg & Sup	With Reg & Sup
SP				
Importance in Signal (↑)	0.11 ± 0.09	0.55 ± 0.30	0.22 ± 0.18	0.54 ± 0.31
Signal/Background (↑)	0.93 ± 0.23	24.27 ± 25.96	2.88 ± 2.69	22.17 ± 21.44
Metric Entropy (True: 0.57)	1.00 ± 0.00	0.73 ± 0.07	0.76 ± 0.08	0.72 ± 0.06
KL Div (↓)	2.57 ± 0.81	0.98 ± 0.85	2.75 ± 1.14	0.99 ± 0.86
TM				
Importance in Signal (↑)	0.05 ± 0.04	0.46 ± 0.19	0.06 ± 0.07	0.50 ± 0.21
Signal/Background (↑)	0.89 ± 0.19	26.13 ± 21.05	1.66 ± 1.72	37.84 ± 34.39
Metric Entropy (True: 0.49)	1.00 ± 0.00	0.75 ± 0.07	0.78 ± 0.08	0.72 ± 0.06
KL Div (↓)	3.26 ± 0.67	1.12 ± 0.96	4.94 ± 1.41	1.12 ± 1.03
MT				
Importance in Signal (↑)	0.16 ± 0.10	0.75 ± 0.18	0.66 ± 0.17	0.76 ± 0.17
Signal/Background (↑)	1.29 ± 0.38	41.98 ± 33.08	23.50 ± 18.12	46.74 ± 43.68
Metric Entropy (True: 0.61)	0.99 ± 0.01	0.70 ± 0.07	0.68 ± 0.07	0.70 ± 0.07
KL Div (↓)	2.08 ± 0.67	0.50 ± 0.48	1.37 ± 0.53	0.50 ± 0.48
CH				
Importance in Signal (↑)	0.30 ± 0.14	0.85 ± 0.15	0.74 ± 0.16	0.85 ± 0.13
Signal/Background (↑)	1.47 ± 0.37	40.98 ± 31.11	15.66 ± 11.27	35.52 ± 25.38
Metric Entropy (True: 0.72)	0.99 ± 0.01	0.77 ± 0.07	0.71 ± 0.06	0.77 ± 0.06
KL Div (↓)	1.34 ± 0.52	0.29 ± 0.39	1.39 ± 0.58	0.31 ± 0.31
TH				
Importance in Signal (↑)	0.40 ± 0.11	0.94 ± 0.06	0.86 ± 0.07	0.95 ± 0.05
Signal/Background (↑)	1.53 ± 0.38	77.01 ± 52.88	18.05 ± 8.63	59.95 ± 40.91
Metric Entropy (0.79)	0.99 ± 0.01	0.80 ± 0.03	0.72 ± 0.07	0.79 ± 0.04
KL Div (↓)	0.95 ± 0.27	0.13 ± 0.16	1.38 ± 0.58	0.24 ± 0.16
NLS				
Importance in Signal (↑)	0.04 ± 0.04	0.16 ± 0.15	0.10 ± 0.10	0.17 ± 0.16
Signal/Background (↑)	1.15 ± 0.27	12.00 ± 15.21	5.69 ± 6.27	16.05 ± 33.60
Metric Entropy (True: 0.38)	0.99 ± 0.01	0.77 ± 0.08	0.79 ± 0.09	0.79 ± 0.07
KL Div (↓)	3.78 ± 0.87	2.62 ± 1.29	3.81 ± 1.08	2.60 ± 1.32
NES				
Importance in Signal (↑)	0.03 ± 0.02	0.03 ± 0.04	0.03 ± 0.07	0.06 ± 0.09
Signal/Background (↑)	0.96 ± 0.33	0.99 ± 1.69	1.17 ± 2.00	2.30 ± 3.15
Metric Entropy (True: 0.42)	0.99 ± 0.01	0.75 ± 0.12	0.80 ± 0.07	0.81 ± 0.06
KL Div (↓)	3.74 ± 0.70	4.48 ± 1.16	5.05 ± 1.49	3.88 ± 1.44
PTS				
Importance in Signal (↑)	0.01 ± 0.01	0.56 ± 0.26	0.47 ± 0.25	0.70 ± 0.28
Signal/Background (↑)	1.46 ± 0.37	335.31 ± 378.09	205.88 ± 249.21	661.50 ± 504.62
Metric Entropy (True: 0.20)	0.99 ± 0.01	0.47 ± 0.15	0.55 ± 0.16	0.39 ± 0.16
KL Div (↓)	4.50 ± 0.50	1.01 ± 1.24	1.60 ± 1.15	0.72 ± 1.05
GPI				
Importance in Signal (↑)	0.25 ± 0.14	0.70 ± 0.14	0.60 ± 0.18	0.63 ± 0.11
Signal/Background (↑)	0.97 ± 0.11	11.46 ± 8.83	6.44 ± 3.70	7.57 ± 5.91
Metric Entropy (True: 0.75)	1.00 ± 0.00	0.82 ± 0.07	0.69 ± 0.09	0.76 ± 0.05
KL Div (↓)	1.55 ± 0.59	0.63 ± 0.50	2.49 ± 0.61	1.85 ± 0.47

Supplementary Table 9. Ablation of loss terms. DCT-based prior regularization and sorting signal supervision are indicated by Reg and Sup in short hand.

	ESM12		ProtT5	
	No Reg & Sup	With Reg & Sup	No Reg & Sup	With Reg & Sup
Predicted Num. Labels	1.28 ± 0.03	1.29 ± 0.02	1.25 ± 0.03	1.26 ± 0.02
Accurary	0.50 ± 0.02	0.51 ± 0.02	0.54 ± 0.02	0.55 ± 0.02
Jaccard	0.65 ± 0.02	0.66 ± 0.02	0.69 ± 0.01	0.69 ± 0.01
MicroF1	0.70 ± 0.02	0.70 ± 0.02	0.73 ± 0.02	0.73 ± 0.01
MacroF1	0.60 ± 0.01	0.62 ± 0.02	0.66 ± 0.01	0.66 ± 0.01
MCC per location (↑ is better)				
Cytoplasm	0.58 ± 0.02	0.59 ± 0.01	0.61 ± 0.01	0.62 ± 0.01
Nucleus	0.63 ± 0.02	0.63 ± 0.02	0.69 ± 0.02	0.69 ± 0.01
Extracellular	0.83 ± 0.03	0.84 ± 0.03	0.83 ± 0.04	0.85 ± 0.04
Cell membrane	0.62 ± 0.02	0.63 ± 0.01	0.66 ± 0.02	0.66 ± 0.01
Mitochondrion	0.72 ± 0.02	0.72 ± 0.03	0.76 ± 0.03	0.76 ± 0.02
Plastid	0.86 ± 0.02	0.87 ± 0.01	0.90 ± 0.02	0.90 ± 0.01
Endoplasmic reticulum	0.47 ± 0.03	0.48 ± 0.03	0.53 ± 0.03	0.56 ± 0.03
Lysosome/Vacuole	0.23 ± 0.04	0.24 ± 0.06	0.30 ± 0.01	0.28 ± 0.04
Golgi apparatus	0.32 ± 0.05	0.30 ± 0.04	0.36 ± 0.05	0.34 ± 0.05
Peroxisome	0.24 ± 0.06	0.41 ± 0.09	0.55 ± 0.06	0.56 ± 0.08

Supplementary Table 10. Results on the SwissProt CV dataset by kingdom. The per-location scores are MCC.

	YLoc+				DeepLoc 1.0			
	Metazoa	Fungi	Viridiplantae	Other	Metazoa	Fungi	Viridiplantae	Other
Accuracy	0.33	0.27	0.31	0.4	0.47	0.43	0.53	0.59
Jaccard	0.52	0.47	0.48	0.55	0.56	0.53	0.59	0.62
MicroF1	0.58	0.55	0.53	0.57	0.58	0.56	0.6	0.63
MacroF1	0.35	0.3	0.38	0.28	0.39	0.38	0.48	0.32
MCC Per location (↑ is better)								
Cytoplasm	0.41	0.32	0.3	0.52	0.44	0.42	0.43	0.6
Nucleus	0.42	0.33	0.51	0.29	0.47	0.37	0.56	0.3
Extracellular	0.65	0.5	0.36	0.7	0.8	0.8	0.48	0.74
Cell membrane	0.48	0.26	0.35	0.23	0.56	0.36	0.48	0.24
Mitochondrion	0.44	0.52	0.46	0.49	0.56	0.61	0.58	0.67
Plastid	0	0	0.66	0	0	0	0.8	0.09
Endoplasmic reticulum	0.18	0.15	0.2	0.05	0.3	0.39	0.38	0.06
Lysosome/Vacuole	0.07	-0.01	0.13	0.08	0.03	0.1	0.09	-0.02
Golgi apparatus	0.11	0.07	0.18	-0.01	0.16	0.16	0.37	-0.01
Peroxisome	0.04	0.04	0.06	0	0.09	0.08	0.28	0.2

	ESM1b				ProtT5			
	Metazoa	Fungi	Viridiplantae	Other	Metazoa	Fungi	Viridiplantae	Other
Accuracy	0.51	0.52	0.58	0.58	0.53	0.53	0.6	0.59
Jaccard	0.67	0.67	0.7	0.67	0.68	0.69	0.71	0.68
MicroF1	0.71	0.73	0.73	0.7	0.72	0.74	0.74	0.71
MacroF1	0.54	0.54	0.63	0.42	0.56	0.55	0.65	0.44
MCC per location (↑ is better)								
Cytoplasm	0.6	0.6	0.57	0.7	0.61	0.61	0.6	0.72
Nucleus	0.65	0.61	0.74	0.37	0.68	0.65	0.77	0.47
Extracellular	0.86	0.88	0.53	0.79	0.86	0.88	0.55	0.79
Cell membrane	0.65	0.47	0.61	0.39	0.67	0.44	0.62	0.28
Mitochondrion	0.7	0.76	0.75	0.66	0.73	0.79	0.74	0.69
Plastid	0	0	0.87	0.2	0	0	0.89	0.2
Endoplasmic reticulum	0.5	0.59	0.46	0.26	0.53	0.6	0.51	0.43
Lysosome/Vacuole	0.21	0.26	0.33	0.31	0.25	0.3	0.35	0.37
Golgi apparatus	0.34	0.29	0.53	0	0.3	0.24	0.59	0
Peroxisome	0.41	0.46	0.58	0.2	0.49	0.55	0.58	0.2
